# Physics of transportation: Towards optimal capacity using the multilayer network framework

**DOI:** 10.1038/srep19059

**Published:** 2016-01-21

**Authors:** Wen-Bo Du, Xing-Lian Zhou, Marko Jusup, Zhen Wang

**Affiliations:** 1 School of Electronic and Information Engineering, Beihang University, Beijing 100191, P.R.China; 2 Faculty of Sciences, Kyushu University, Fukuoka 819-0395, Japan; 3 Interdisciplinary Graduate School of Engineering Sciences, Kyushu University, Fukuoka 816-8580, Japan

## Abstract

Because of the critical role of transportation in modern times, one of the most successful application areas of statistical physics of complex networks is the study of traffic dynamics. However, the vast majority of works treat transportation networks as an isolated system, which is inconsistent with the fact that many complex networks are interrelated in a nontrivial way. To mimic a realistic scenario, we use the framework of multilayer networks to construct a two-layered traffic model, whereby the upper layer provides higher transport speed than the lower layer. Moreover, passengers are guided to travel along the path of minimal travelling time and with the additional cost they can transfer from one layer to another to avoid congestion and/or reach the final destination faster. By means of numerical simulations, we show that a degree distribution-based strategy, although facilitating the cooperation between both layers, can be further improved by enhancing the critical generating rate of passengers using a particle swarm optimisation (PSO) algorithm. If initialised with the prior knowledge from the degree distribution-based strategy, the PSO algorithm converges considerably faster. Our work exemplifies how statistical physics of complex networks can positively affect daily life.

Statistical physics of complex networks^1^, due to broad applicability, has served as the analytical foundation for the analysis of many problems related to the modern infrastructure^2^,^3^,^4^,^5^,^6^. Physics of transportation in particular received a lot of attention^7^,^8^,^9^,^10^,^11^,^12^,^13^, which may not come as a surprise given the increasingly severe traffic congestion around the world. For achieving a higher capacity, it was shown that both the network structure and the routing strategy are of major importance when designing vital transportation infrastructure^14^,^15^,^16^,^17^,^18^,^19^. Accordingly, the number of routing algorithms based on physical methods has swelled over the years, where some of the better known examples include global dynamic routing^16^, degree-driven delivering strategy^17^, betweenness related edge deletion^18^, traffic-awareness strategy^20^,^21^, adaptive routing strategy^22^, and the optimal local routing strategy^23^, to name a few.

Despite all the interest in physics of transportation, a prevailing assumption has been that the nodes of a network have a single degree distribution and therefore interact in a manner described by a single network layer. In reality, however, most transportation systems are composed of many interconnected networks, thus representing a typical problem for which statistical physicists devised the multilayer network framework^24^,^25^,^26^,^27^,^28^,^29^,^30^,^31^. Within such a framework, many interesting dynamical phenomena may arise. One of the best known examples is a cascading failure of interdependent networks, whereby seemingly irrelevant alterations in one network have unexpected and catastrophic consequences for the other^24^. Thereafter, physicists in collaboration with scientists from related disciplines examined the multilayer network structure in the context of (i) assortativity and the robustness against attacks^32^, (ii) inter-similarity and cascading failure^33^, (iii) diffusion^34^, (iv) disease spreading and prevention^35^, (v) evolutionary games^36^,^37^, (vi) voting^38^, and (vii) synchronisation^39^. In some instances, physics of transportation either motivated or was the direct target of research based on multilayer networks^40^,^41^. A particularly nice example are interrelated networks of airports and seaports such that, in a given city, the functioning of the city’s airport is dependent upon resources coming from the seaport and vice versa^42^.

The multilayer framework, as it appeared in statistical physics of complex networks, promises to describe traffic dynamics beyond what is possible using more traditional isolated networks. By representing physical infrastructure as one network layer and traffic flows as another, ref. 31 found some fundamental differences between the load estimators and the real load. Furthermore, a model of layered transportation in ref. 27 led to the introduction of the cooperation strength as an order parameter and revealed a phase transition at the onset of cooperation. The order parameter changed from zero to positive at the transition point. Finally, the results of ref. 28 indicated that coupled spatial systems may cause behaviour dependent on the interplay between the coupling and the source-sink distribution. Inspired by these discoveries, we use a two-layered network model to optimise the capacity of a predetermined infrastructure, where a central role is played by transfer costs incurred when passengers switch between the coupled networks. We show that an optimised allocation of transfer costs is achievable using a particle swarm optimisation (PSO) algorithm to increase the total capacity of the infrastructure.

## Results

**Figure 1:**
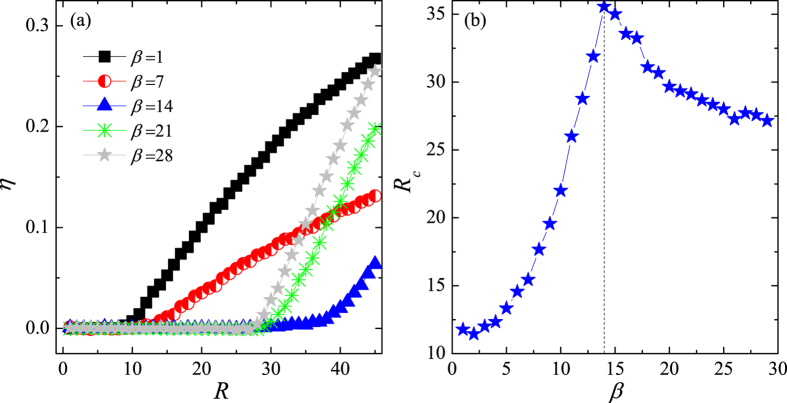
System’s performance under an even transfer-cost allocation. (**a**) Order parameter *η* as a function of generating rate *R* at several values of transfer cost *β*. (**b**) Critical generating rate *R*_*c*_ as a function of *β*. Maximum *R*_*c*_ corresponds to *β* = 14 marked by the dashed line. The results are averaged over 30 independent realisations.

We start the analysis of the model described in the Methods section from the simplest possible case—a constant transfer cost, *β*, at all coupled nodes (i.e. *β*_*p*_ = *β*, where *p* indexes a unique node). Let us denote by *R* the number of new passengers that enter the system at each time step *t*. Source and destination locations of passengers are assigned randomly. Furthermore, nodes are assumed to have the capacity to deliver *C* passengers in a single time step. One of the most important properties of a transportation system is the balance between passenger handling and delivering capacities, which is appropriately captured by the critical generating rate, *R*_*c*_, at which a continuous phase transition takes place from a free-flow to a congested state. To describe such a critical phenomenon, we consider an order parameter, *η*^14^,^43^, defined as


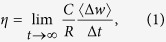


where 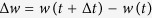, with *w*(*t*) being the total number of passengers in the system at time step *t*, and 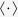 the averaging operator over time windows of width Δ*t*. In a free-flow state (i.e. *R* < *R*_*c*_), the balance between generated and removed traffic guarantees *η* = 0. By contrast, a congested state (i.e. *R* > *R*_*c*_) occurs if the number of accumulated passengers increases with time, thus causing *η* > 0. Figure 1(a) shows order parameter *η* as a function of *R* at different values of the transfer cost, *β*. We see that *η* = 0 when *R* is small irrespective of the value of *β*, but begins to increase monotonously when *R* reaches the critical point, *R*_*c*_. Moreover, because the transition happens at different critical points, the network capacity varies with *β*. An interesting question thus poses itself; what value of the transfer cost, *β*, maximises the system’s capacity?

The simulation results in Fig. 1(b) show that *β* = 14 maximises the critical threshold *R*_*c*_. If the transfer cost is low (e.g. *β* = 1), passengers prefer to travel in the upper layer due to its high speed, but can easily return to the lower layer to reach their final destinations—a form of cooperation establishes itself between the two layers. However, as passengers enter the system at a higher and higher rate, the upper layer reaches a congestion state, which in turn causes a system-wide traffic jam. The system’s capacity is thus close to the capacity of the upper layer. By contrast, if the transfer cost is excessive (e.g. *β* = 29), most passengers choose to stay only in one layer—it becomes more difficult to establish the same form of cooperation as before. Because the lower layer has a greater capacity, the total system’s capacity approaches that of the lower layer. Between these extremes (e.g. *β* = 14), high speed and extra transfer costs are well-balanced to produce the optimal capacity of the system.

**Figure 2:**
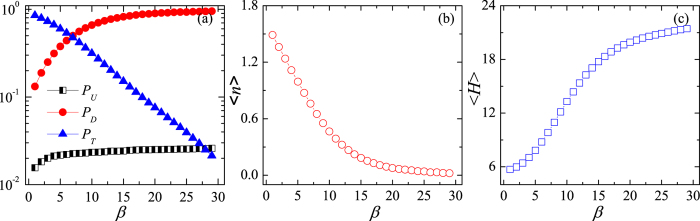
How the optimal capacity arises? (**a**) Traffic flows *P*_*U*_, *P*_*D*_, and *P*_*T*_, (**b**) the average number of transfers, 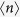, and (**c**) the average number of hops, 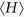, all presented as the functions of transfer cost *β*.

To better understand how the optimal capacity arises, we investigate the dependence of the travel behaviour of passengers on the transfer cost. Specifically, the traffic flow is subdivided into three types: the flow in the upper layer, *P*_*U*_, the flow in the lower layer, *P*_*D*_, and the flow of passengers transferring from one layer to another, *P*_*T*_. As shown in Fig. 2(a), *P*_*T*_ decreases, while *P*_*U*_ and *P*_*D*_ increase with *β*, thus indicating that passengers are more bound to a layer where they entered the system when the cost is high. This result is further confirmed by the average number of transfers 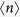, which monotonously decreases with *β* as shown in Fig. 2(b). A higher transfer cost, therefore, suppresses the cooperation between the two layers. Suppressed cooperation diminishes the opportunities for exploiting more direct routes in the upper layer and hence passengers have to endure stopping at more stations, which is indicated in Fig. 2(c) by the average number of hops along the way, 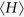. When the system is functioning at the maximum capacity (i.e. *β* = 14), we have 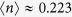 and 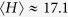, suggesting more reliance on the lower layer with only a limited number of passengers transferring between layers to alleviate congestion.

Thus far, we explored the simplest assumption of an even distribution of the transfer cost (i.e. *β*_*p*_ = *β* for all *p*), whereas in reality the transfer cost may have different values in each coupled node. Inspired by the knowledge that the optimal capacity is often related with degree centrality or betweenness centrality^14^,^17^, we introduce a new transfer cost as:


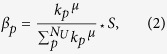


where *k*_*p*_ is the degree of coupled node *p* in the upper layer, *S* = *β* * *N*_*U*_ is the total cost of transfers  *N*_*U*_ is the number of nodes in the upper layer, and *μ* is an adjustable parameter. If *μ* = 0, we recover the above-studied case of an even transfer-cost allocation (hereafter “EFA”); otherwise the allocation is biased (“BFA”).

**Figure 3:**
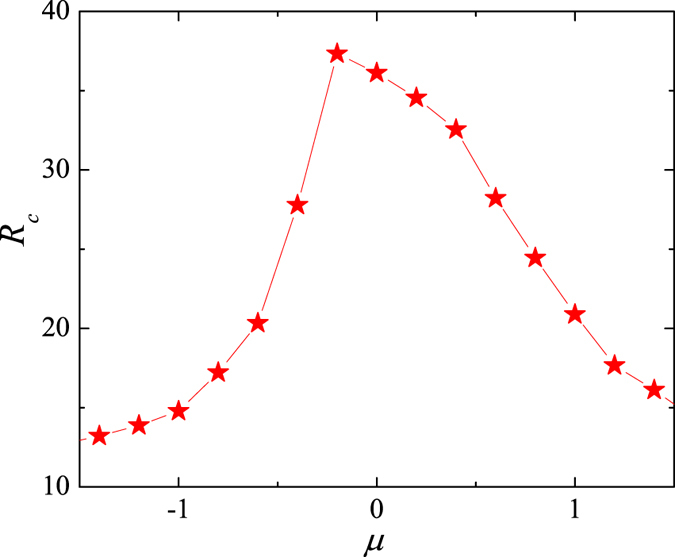
Optimising the system’s capacity under a biased transfer-cost allocation. Critical generating rate *R*_*c*_ as a function of parameter *μ* at *S* = 2800 (corresponding to *β* = 14).

**Figure 4:**
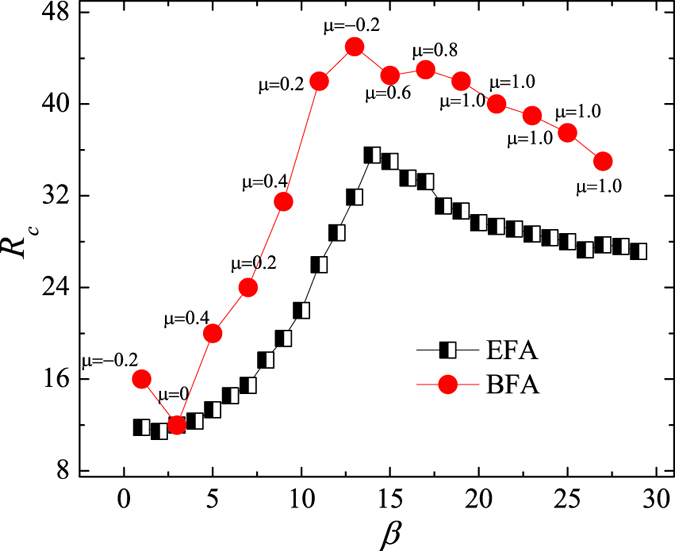
Comparing the two transfer-cost allocations (EFA and BFA). Critical generating rate *R*_*c*_ as a function of transfer cost *β*. The optimal value of parameter *μ* changes with *β*.

In analysing the BFA, we first explore if there exists a particular value of *μ* that maximises the system’s capacity at fixed *β*. In Fig. 3, the critical point, *R*_*C*_, is shown as a function of *μ*, where *S* = 2800 to maintain *β* = 14 from Fig. 1(b). The maximum value of *R*_*c*_, and hence the optimal capacity, is indeed reached at *μ* = −0.2. Yet, when *β* changes, Fig. 4 indicates that reaching the maximum capacity requires *μ* to change as well. The failure to find a single best value of *μ* seems to be pointing to the conclusion that the allocation of transfer costs according to degree centrality may not be the best strategy.

If degree centrality is not the best strategy, can we find a better transfer-cost allocation that would make the system’s capacity higher? The problem can formally be written as


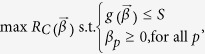


where 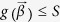 is the inequality constraint, with 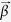 being a short-hand notation for a vector comprised of components 
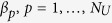
. We set 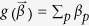 to define an upper limit, *S*, for the total transfer cost. The critical generating rate of passengers, 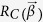, is in the context of optimisation called the objective function. Vector 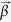 that maximises the objective function and satisfies the above-defined inequality constraint and variable bounds is called a feasible solution; otherwise the solution is infeasible.

Because the objective function, 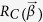, is nonlinear and without an analytical expression in the case of coupled networks, we resort to intelligent computing to solve the optimisation problem at hand. Specifically, we employ the particle swarm optimisation (PSO) algorithm^44^, a technique inspired by the social behaviour of swarms^45^ and widely used to solve a number of optimisation problems^46^,^47^,^48^,^49^,^50^. The PSO algorithm with constraint handling used herein searches for a solution while obeying the following rules:

1. The objective function is redefined and separated into two parts: the fitness function, 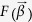, and the constraint violation function, 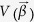, where 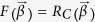 and 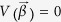 if 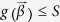; otherwise 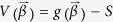.
2. A pair-wise comparison of candidate solutions is performed in such a way that:

- when two feasible solutions are compared, the one with higher fitness is chosen;
- when two infeasible solutions are compared, the one with lower constraint violation is chosen;
- when a feasible and an infeasible solutions are compared, the infeasible solution is chosen only if it has higher fitness and the constraint violation is less than *ε* > 0; otherwise the feasible solution is chosen.

As a result of these rules, the PSO algorithm may choose an infeasible solution as the optimum, but such a solution is guaranteed to be near the boundary of the set of feasible solutions.

**Figure 5:**
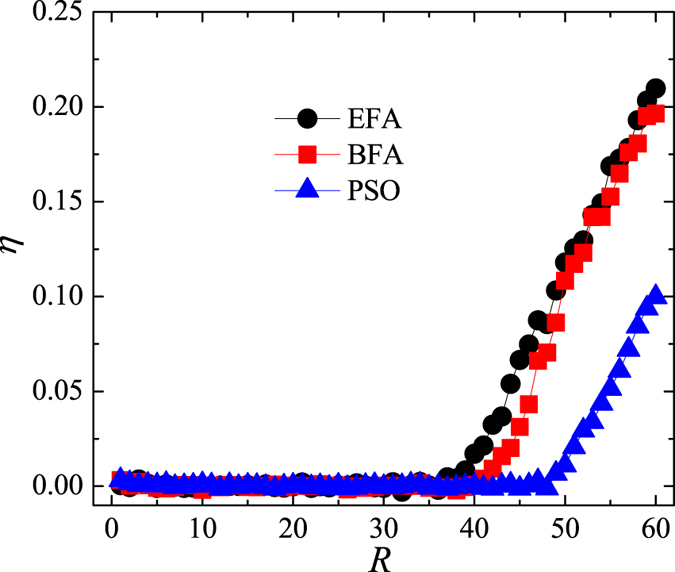
Particle swarm optimisation increases the system’s capacity. Order parameter *η* as a function of generating rate *R* under the three transfer-cost allocations (EFA, BFA, and PSO).

**Figure 6:**
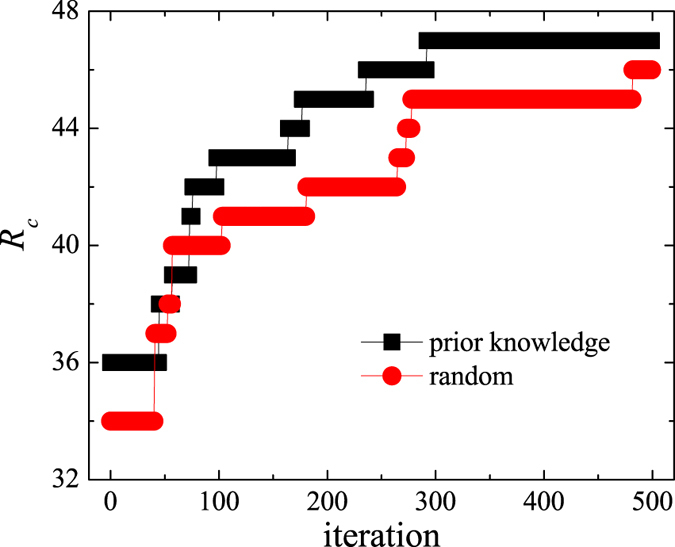
Improving the performance of the PSO algorithm. Critical generating rate *R*_*c*_ as a function of the number of iterations under the two initialisation methods (random and prior knowledge).

Next, we examine the performance of our toy-model transportation system after optimisation with the PSO algorithm. Figure 5 displays the order parameter, *η*, as a function of the passenger generating rate, *R*, under different transfer-cost allocation methods. The PSO algorithm considerably increases the critical point, *R*_*c*_, relative to EFA and BFA methods, thus clearly outperforming them. A drawback of the PSO algorithm is a lengthy computation time, especially if the algorithm is initialised randomly. From Fig. 3, we known that the maximum *R*_*c*_ is obtained for *β* = 14 and *μ* = −0.2, so it seems reasonable to use such prior knowledge during initialisation to decrease the computation time. Figure 6 compares the performance of the PSO algorithm under the two initialisation options. If the prior knowledge is used, the PSO algorithm converges faster and is able to find a better solution (i.e. higher *R*_*c*_) within the preset number of iterations. We, therefore, discuss the properties of the optimised transportation system only in the case of initialisation with the prior knowledge.

**Figure 7:**
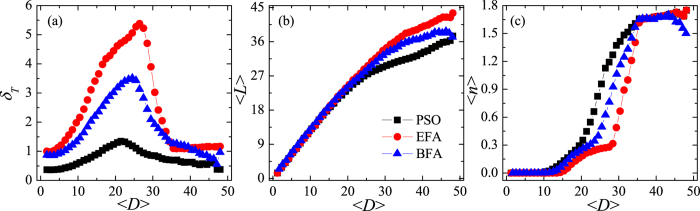
Performance of the optimised system. (**a**) The variance of the travelling time, *δ*_*T*_, (**b**) the average cost of traversed paths, 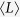, and (**c**) the average number of transfers, 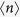, as functions of average distance 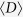 between departure and destination nodes. The generating rate is *R* = 60.

A closer look at the performance of the PSO algorithm as a function of the (Euclidean) distance, 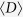, between departure and destination nodes reveals several interesting outcomes. Here, as in refs 51,52, the variance of the travelling time, *δ*_*T*_, is used as an intuitive reliability indicator because it is highly undesirable for some passengers to be stuck, while others progress smoothly through the system. Figure 7(a) shows that the PSO algorithm controls *δ*_*T*_ much better than EFA or BFA methods, indicating that the optimised system functions more reliably. Furthermore, in Fig. 7(b) the average cost of traversed paths, 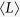, is lower if the system is optimised using the PSO algorithm instead of EFA or BFA methods. The last result, to be precise, holds only if 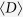 is higher than 15, but mid- to long-distance journeys are the ones where cutting costs makes the most economic and ecological sense (e.g. by considerably reducing the fuel consumption). Finally, Fig. 7(c) shows that in the interval 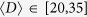 the PSO algorithm generates the highest average number of transfers, 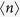, of the three methods. Optimised systems, therefore, are more reliant on transferring passengers who travel mid to long distances in order to alleviate congestion.

## Conclusion

Using ideas and concepts from statistical physics of complex networks, particularly the multilayer network framework, we built a two-layered idealised transportation system and examined the implied dynamics. Each layer had a different topology and supported different travelling speed—characteristics that qualitatively correspond to real-life transportation systems. Seeking to minimise the travelling time of passengers, we allowed transfers between the two layers at an extra cost with the purpose of (i) taking the advantage of high speed offered by the upper layer or (ii) reaching a final destination served only by the, in relative terms slow, lower layer.

Our main concern was the influence of transfer costs on the transportation system’s capacity. We found that an even transfer-cost allocation (EFA) maximises the system’s capacity by fine-tuning the trade-off between high speed and extra transfer costs. Somewhat at odds with the general consensus reached by examining traffic dynamics in isolated networks, a degree centrality-based strategy (BFA) was not overly helpful in enhancing the performance of the system. However, starting from such a strategy and reassigning transfer costs using a particle swarm optimisation (PSO) algorithm improved the capacity and several other properties of the system at a reasonable computational cost. Our work exemplifies how statistical physics of complex networks can positively affect daily life.

Finally, a word of caution regarding the optimality of the solution is in order. When considering real-world optimisation problems, it is often hard, if not impossible, to obtain a theoretical optimal solution. Therefore, it is quite common to rely on heuristic optimisation—such as the genetic algorithm, differential evolution, and PSO—although none of these methods guarantees an optimal solution. What heuristic optimisation usually does is improving upon a solution suggested by experts. Herein, for example, we combine the commonly used and well-documented PSO algorithm with a strategy suggested by the system’s topology in order to allocate transfer costs “optimally”. The main contribution thus is precisely the fact that we *can* find an optimisation-based strategy that is better than the topology-based (i.e. “expert”) strategy. We stayed clear of comparing the current best solution with the best solutions that other algorithms may have generated, the reason being that such a comparison is perhaps more interesting to the readers of journals specialised in intelligent computing. Nevertheless, it is an interesting topic for future research.

## Methods

Here, we consider a toy model that exhibits the same main characteristics as many modern transportation systems composed of, for example, a train and an aeroplane network. The model employs a two-layer architecture: the lower layer is a von Neumann-neighbourhood lattice containing *N*_*D*_ × *N*_*D*_ nodes^53^, whereas the upper layer, constructed using the scale-free algorithm^54^, has *N*_*U*_ nodes with the average degree 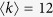. The transport speed in each network is different. Without any loss of generality, we work in units in which the speed of the lower layer is *V*_*D*_ = 1 and the speed of the upper layer is *V*_*U*_ = *α* × *V*_*D*_, where we fix *α* = 3 as the realistic speed ratio between aeroplane and high-speed railway networks. To make transfers between the two layers possible, each node in the upper layer is connected to one randomly selected counterpart from the lower layer (i.e. there are *N*_*U*_ coupled nodes). For the purpose of calculating distances, nodes in the upper layer are assigned the same coordinates as their counterparts from the lower layer. Furthermore, the taxicab metric is used in the lower layer, where the length of an edge connecting two neighbours is *D*_*ij*_ = 1. In the upper layer, by contrast, the distance between two neighbours is given by the Euclidean metric, 
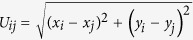
, where (*x*_*i*_, *y*_*i*_) are the coordinates of node *i*. For simplicity, we mainly focus on two layers with *N*_*D*_ = 200 and *N*_*U*_ = 35. Enlarging both networks by the same factor guarantees the same quantitative observations.

After defining the underlying infrastructure, the transport process is carried out as follows. At each time step, *t*, a number of new passengers, *R*, with random sources and destinations are assigned to the system of networks. Each node has the capacity to deliver *C* passengers in one time step (we set *C* = 1 in simulations), complemented with an infinite queue buffer to handle the congestion. Passengers in the system are navigated in the way that minimises their travelling time. The primary purpose of transfers is to reduce the total time of travel, which comes at an economic cost. Generally, a passenger is willing to pay a certain number of monetary units for a given time reduction, thus allowing us to express the transfer cost, *β*_*p*_, at coupled node *p* in units of time. With these definitions, passengers between any two nodes *i* and *j* are delivered along such a path, 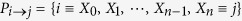, that minimises the quantity


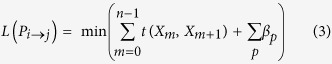


where the first term in brackets is the total time of travel, given here as the sum of individual travelling times 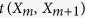 between the two successive nodes, *X*_*m*_ and *X*_*m*+1_, along path *P*_*i*→*j*_ calculated as the ratio of speed to distance. The second term, 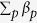, represents the total transfer cost. When a passenger reaches the destination, it is removed from the system. Note that quantity *L* can be interpreted as the total cost in units of time associated with path *P*_*i*→*j*_.

The PSO algorithm is usually specified by three parameters. We denote these parameters with *c*_1_, *c*_2_, and *η*. The values are *c*_1_ = *c*_2_ = 2.05 and *η* = 0.7298. The number of particles is 20 and the number of iterations 300.

## Additional Information

**How to cite this article**: Du, W.-B. *et al.* Physics of transportation: Towards optimal capacity using the multilayer network framework. *Sci. Rep.* **6**, 19059; doi: 10.1038/srep19059 (2016).
